# Engineer Nanoscale Defects into Selective Channels: MOF-Enhanced Li^+^ Separation by Porous Layered Double Hydroxide Membrane

**DOI:** 10.1007/s40820-023-01101-w

**Published:** 2023-06-07

**Authors:** Yahua Lu, Rongkun Zhou, Naixin Wang, Yuye Yang, Zilong Zheng, Miao Zhang, Quan-Fu An, Jiayin Yuan

**Affiliations:** 1 Beijing Key Laboratory for Green Catalysis and Separation, Department of Chemical Engineering, Beijing University of Technology, Beijing, 100124 People’s Republic of China; 2 Faculty of Materials and Manufacturing, Beijing University of Technology, Beijing, 100124 People’s Republic of China; 3 Department of Materials and Environmental Chemistry, Stockholm University, 10691 Stockholm, Sweden

**Keywords:** Nanoscale defect construction, Nanoparticles restrict growth, Two-dimensional composite membrane, Lithium-ion extraction, High stability

## Abstract

Two-dimensional (2D) membrane-based ion separation technology has been increasingly explored to address the problem of lithium resource shortage, yet it remains a sound challenge to design 2D membranes of high selectivity and permeability for ion separation applications. Zeolitic imidazolate framework functionalized modified layered double hydroxide (ZIF-8@MLDH) composite membranes with high lithium-ion (Li^+^) permeability and excellent operational stability were obtained in this work by in situ depositing functional ZIF-8 nanoparticles into the nanopores acting as framework defects in MLDH membranes. The defect-rich framework amplified the permeability of Li^+^, and the site-selective growth of ZIF-8 in the framework defects bettered its selectivity. Specifically speaking, the ZIF-8@MLDH membranes featured a high permeation rate of Li^+^ up to 1.73 mol m^−2^ h^−1^ and a desirable selectivity of Li^+^/Mg^2+^ up to 31.9. Simulations supported that the simultaneously enhanced selectivity and permeability of Li^+^ are attributed to changes in the type of mass transfer channels and the difference in the dehydration capacity of hydrated metal cations when they pass through nanochannels of ZIF-8. This study will inspire the ongoing research of high-performance 2D membranes through the engineering of defects.

**Supplementary Information:**

The online version contains supplementary material available at 10.1007/s40820-023-01101-w.

## Highlights

- The zeolitic imidazolate framework functionalized modified layered double hydroxide (ZIF-8@MLDH) composite membranes with superior structural stability and Li^+^ permeability are prepared by selectively growing ZIF-8 nanoparticles in the framework defects of the MLDH membrane.
- The tailor-made ZIF-8@MLDH membrane has a large Li^+^ permeability of up to 1.73 mol m^-2^ h^-1^ and a high Li^+^/Mg^2+^ selectivity of 31.9, which exceed most of the current 2D lamellar membranes.

**Supplementary Information:**

The online version contains supplementary material available at 10.1007/s40820-023-01101-w.

## Introduction

The demand for lithium recently has been growing rapidly worldwide [1–3]. Extracting Li^+^ from salt-lake brines is increasingly recognized as one of the most important source of lithium, efficient extraction of lithium from salt lake brine is an inevitable choice for sustainable development of lithium industry. However, most brines comprise a high concentration of magnesium ion (Mg^2+^) yet a comparably much low concentration of Li^+^, which hampers the utilization of lithium resources. The primary concern of extracting Li^+^ from salt-lake brines is the insufficiently selective separation of Li^+^ from Mg^2+^ [4–6]. Among the state-of-the-art separation approaches, the membrane technology stands out for promising Li^+^/Mg^2+^ separation [7–9]. Nevertheless, the development of Li^+^/Mg^2+^ separation membranes suffers from a renowned trade-off between permeability and selectivity [10]. In this context, the exploration of new materials and optimized membrane microstructures are key to breaking the trade-off and improving separation performance.

2D materials, which are single or few atomic layers in thickness, have been widely used to prepare membranes with enhanced separation performance [11–13]. The nanoscopic interlayer spacing can serve as fast and selective transport channels to break through the upper limit of traditional membranes in ion sieving [14, 15]. However, the horizontal transport channels are invalid to the overall permeability of the membrane. The tortuous and lengthy transport channels impede further elevation in permeability. To shorten the transmembrane transport distance, the 2D sheets have been reported to carry nanosized holes [16, 17], to boost water flux by three orders of magnitudes in comparison to the aquaporin channels; yet the rejection of the membrane for ions dropped to 20–60%, which fails to meet the requirement in Li^+^/Mg^2+^ separation. As mitigation, the obtained nanoholes as defects on the nanosheets can be patched by nanoparticles to raise selectivity with a little-to-no sacrifice of permeability.

Metal–organic framework (MOF)-based materials have attracted extensive attention in ion separation techniques, especially in Li^+^ extraction due to high selectivity [18, 19]. For instance, Xu and co-workers used MOF-based membranes with dense pores of subnanometer size to achieve an ultrahigh Li^+^ permeance [20]. However, pure MOF-based bulk membranes suffer from the weak stability issue in an aqueous environment that degrades the separation performance in a short term. Inspired by previous studies, the idea of positioning MOF nanoparticles locally inside the 2D framework defects shows up as an attractive and rational strategy for us to stabilize MOF structures by nanoconfinement and thus transfer nanopores of membranes as frame defects into selective channels for Li^+^.

**Fig. 1:**
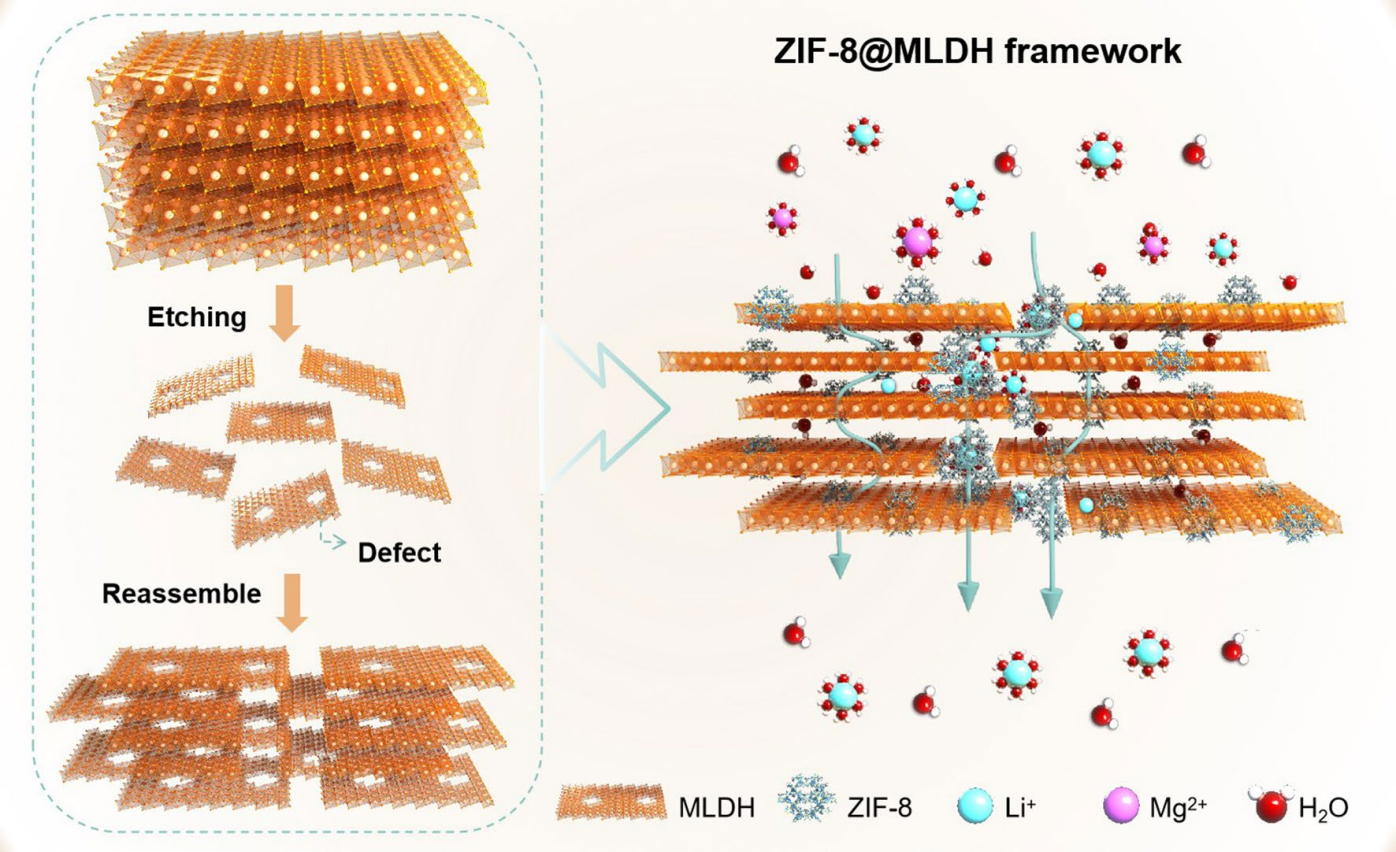
Sketch of the preparation procedure and the transport nanochannels for Li^+^ in ZIF-8@MLDH framework membranes

Herein, we developed a zeolitic imidazolate framework functionalized modified layered double hydroxide (ZIF-8@MLDH) composite membranes bearing fast and selective Li^+^ transport nanochannels. LDH is considered a promising 2D membrane material due to the advantages of easy preparation and low cost. Moreover, LDH nanosheet has the chemical property of bimetal composition, by adjustment of which a high-density mass transfer channels can be accessed. In view of the desirable properties of LDH materials and the controllability of the layer atoms, LDH nanosheets are considered promising materials for the preparation of 2D membranes [21–24]. To our best knowledge, it is the first attempt at the separation of Li^+^/Mg^2+^ by LDH membranes. As shown in Fig. 1, to shorten the transport path of Li^+^ and water molecules, the LDH was transformed into oligolamellar nanoporous MLDH nanosheets by a one-step alkaline etching treatment (OH^−^ + Al(OH)_3_ = AlO_2_^−^ + 2H_2_O). The defect-rich MLDH nanosheets with tiny nanoholes were reassembled into a framework, where the formed nanoholes as defects could minimize the horizontal transport distance and amplify the vertical mass flow. To note, transport channels formed by these pore-like defects without modification were non-selective for Li^+^ and Mg^2+^. This issue was resolved here by in situ growth of ZIF-8 nanoparticles on the defects to raise the selectivity of Li^+^/Mg^2+^ through the aperture of ZIF-8. The as-developed ZIF-8@MLDH composite membranes satisfied both fast transports of Li^+^ with high selectivity against Mg^2+^, K^+^, and Na^+^ due to the combined effects of size exclusion and binding affinity.

## Experiment Section

### Materials

The chemicals were reagent grade or higher purity. Deionized water was used throughout the experiments. Mg(NO_3_)_2_·6H_2_O, Al(NO_3_)_3_·9H_2_O, hexamethylene etamine, potassium hydroxide, Zn(NO_3_)_2_, dimethylimidazole, ethanol, and NH_3_·H_2_O were purchased from Aldrich Co. and used without further purification. In addition, MgCl_2_, LiCl, KCl, and NaCl were purchased from Tianjin Jinteng Co.

### Preparation of ZIF-8@MLDH Membranes by Modified LDH Nanosheets

#### Synthesis of MLDH Nanosheets

The MgAl-LDH powder was synthesized by the procedure reported previously [25]. Mg-Al LDH powder (0.1 g) was immersed in KOH solution (80 mL, 1.0 M) in a Teflon-lined stainless steel autoclave with a capacity of 100 mL. The autoclave was sealed and heated at 160 °C for 20 h [23]. After the end of the reaction, the solution was removed from the Teflon lined and repeatedly washed and centrifuged with deionized water until the solution was weakly alkaline. Then, the MLDH solution was used to prepare the MLDH membrane. The concentration of the obtained MLDH solution was ∼0.089 mg mL^−1^.

#### Preparation of LDH Membrane and MLDH Membrane

The layered LDH and MLDH nanosheets were deposited on the substrate by vacuum-assisted filtration method to obtain the initial LDH and MLDH membranes. The deposition amount of LDH and MLDH nanosheets were both ∼195 mg m^−2^. It is worth noting that the layered LDH nanosheets were prepared according to the reported literature [26]. The thickness of the MLDH membrane can be adjusted by the amount of nanosheet loaded. In this study, the load capacity of the LDH membrane and MLDH membrane was the same.

#### Preparation of ZIF-8@MLDH Membranes

Zinc nitrate and dimethyl imidazole solutions in a defined concentration in ethanol solution were permeated through MLDH membrane by vacuum filtration at a low pressure of 0.1 MPa, and the mixture was then air dried at room temperature to obtain precursor@MLDH membrane. The dry precursor@MLDH membrane was then soaked in a mixture solution of ethanol /NH_3_·H_2_O (v/v) at room temperature for 12 h. Finally, the ZIF-8@MLDH membrane was obtained by drying at 40 °C for 12 h.

## Results and Discussion

### Synthesis and Characterization of the MLDH Nanosheets and MLDH Membranes

**Fig. 2:**
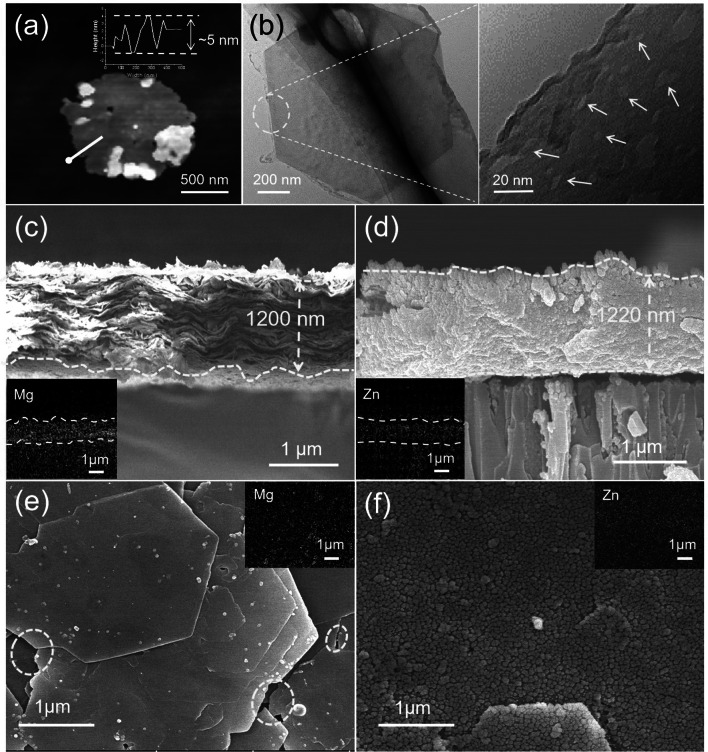
**a** AFM and **b** TEM images of the MLDH nanosheet and its high-magnification view. **c** Cross-sectional and **e** surface SEM images of the MLDH membrane. The inset shows the EDS mapping of Mg. **d** Cross-sectional and **f** surface SEM images of the ZIF-8@MLDH membrane. The inset shows the EDS mapping of Zn

As the building units, MLDH nanosheets were prepared by one-step alkaline etching of LDH bulk (Fig. S1). The X-ray diffraction (XRD) analysis indicated successful exfoliation of MLDH nanosheets. However, the characteristic diffraction peak of the LDH nanosheets exfoliated by the traditional solvent stripping method also disappeared (Fig. S2). The obtained MLDH nanosheets in a hexagonal form were measured to be 5 ± 1 nm in thickness by atomic force microscopy (AFM, Fig. 2a) and 1.5 ± 0.5 μm in lateral size by transmission electron microscopy (TEM, Fig. 2b), which suits well the construction of uniform and ordered laminar thin membranes. The average pore size of the defects on the MLDH nanosheet was measured as 16 ± 2 nm, i.e., nano defects, which was consistent with the gas sorption results (Fig. S3). Both LDH and MLDH-based membranes were prepared by vacuum filtration of their respective aqueous dispersions onto a porous AAO substrate. To avoid damage to the substrate by the strong alkaline solution, the MLDH dispersion was washed till pH = 8.5 before filtration. Note that the MLDH nanosheets were positively charged at this pH (Fig. S4) with a Zeta potential value of + 32.7 mV. This value increases marginally under neutral conditions, which was conducive to the separation of Li^+^/Mg^2+^. The scanning electron microscope (SEM) images verified a typical layered stacking morphology of the MLDH membrane of 1.2 ± 0.1 μm in thickness (Fig. 2c, e). It can be seen from the energy-dispersive spectrometer (EDS) that the characteristic elements of MLDH are evenly distributed along the cross-sectional and on the surface (Insert map in Fig. 2c, e). The interlayer spacing of the MLDH membrane was measured to be 0.82 ± 0.03 nm, which matches well that of the LDH membrane (Fig. S5).

To note, the framework defects in the pristine LDH membrane and the MLDH membranes differ, as detected by low-field nuclear magnetic resonance (NMR) spectroscopy using water as a probe molecule (Fig. S6). The transverse relaxation time (T_2_) can be usually used to characterize the microstructure of the membrane. The wide population in the time domain of 10^3^–10^4^ ms indicates the presence of free water molecules passing through the membranes (i.e., free space). In the time domain of 10–10^3^ ms, the peak corresponding to the framework defects emerges due to holes existing in the disordered stacking of nanosheets and holes created by the alkaline etching process. The increased relaxation time in this region corroborates an increase in the quantity and size of the defects in the MLDH than in the LDH framework. Moreover, the relaxation time in the domain of 0.1–10 ms standing for the interlayer spacing was unchanged, implying the interlamellar structure of LDH and MLDH membranes was similar, which agrees with the XRD analysis (Fig. S5).

### Characterization of ZIF-8@MLDH Membranes

**Fig. 3:**
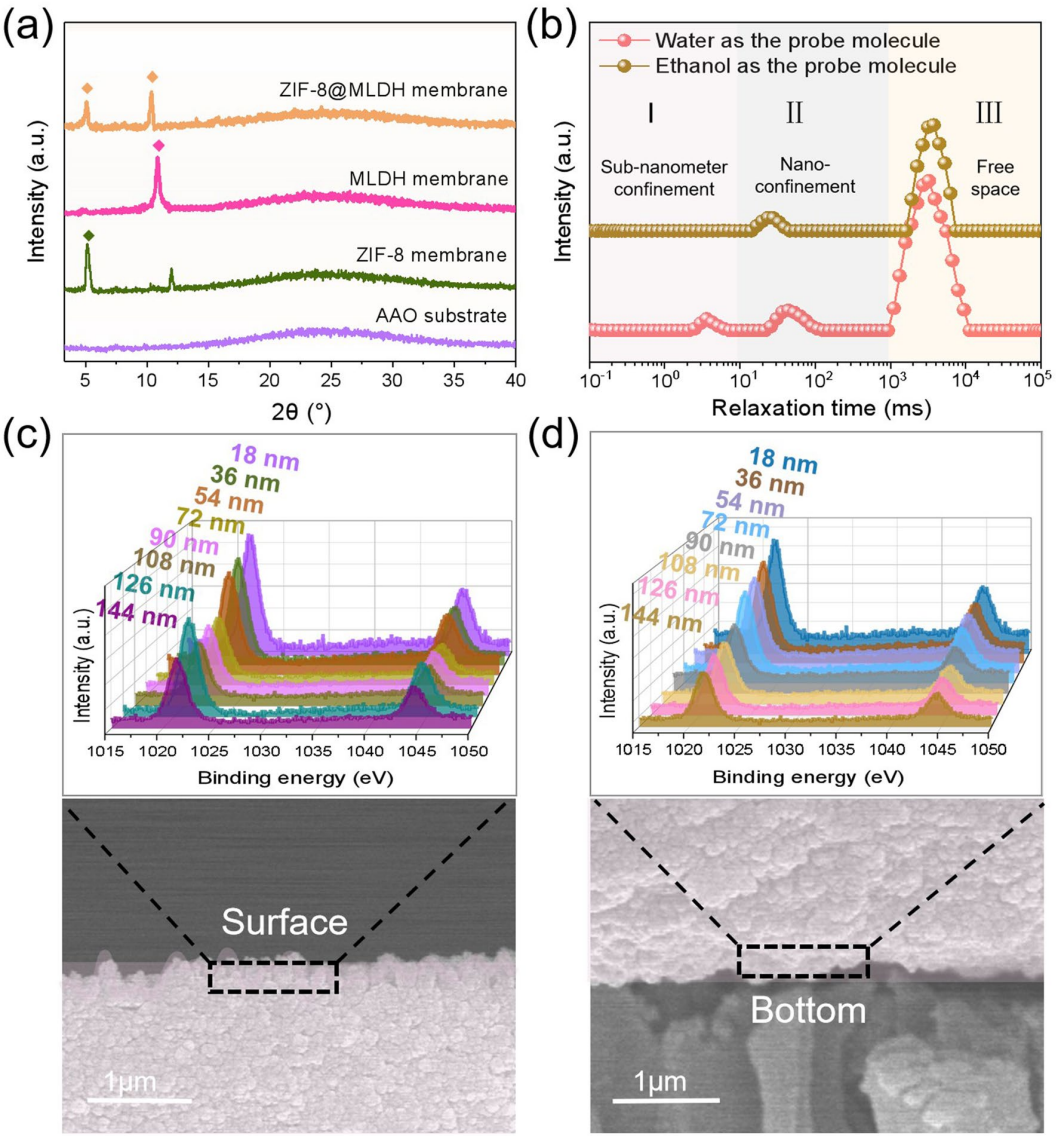
**a** XRD diagrams of the AAO substrate and membranes prepared under different conditions. **b** ^1^H time-domain NMR spectra of MLDH membrane with water and ethanol as the probe molecule. **c** Upper and **d** bottom surfaces of the ZIF-8@MLDH membrane and the corresponding high-resolution XPS spectra of Zn element at different depths

When ZIF-8 nanoparticles were in situ grown in the MLDH framework defects, the pin holes were filled by nanoparticles, as shown in Fig. 2d, f. It can be seen from the high-magnification SEM cross section (Fig. S7) that the size of the nanoparticles is about 30 ± 3 nm. In the EDS spectrum as the inset, the distribution of characteristic elements Zn of ZIF-8 is uniform along the cross-sectional and on the surface. The successful growth of ZIF-8 in the MLDH membrane was supported by XRD analysis that shows the characteristic diffraction peaks of the ZIF-8 phase in the ZIF-8@MLDH membrane (Fig. 3a). Contradictorily, the thickness of the MLDH membrane remained generally unchanged, as most of the nanoparticles grew in situ inside the MLDH framework with only a trace amount on the outer surface (Fig. 2d). The ZIF-8@MLDH composite membrane was readily peeled off from the substrate and next subject to X-ray photoelectron spectroscopy (XPS) analysis to approach the spatial distribution of ZIF-8 nanoparticles. The upper and bottom surfaces of the ZIF-8@MLDH membrane were freshly etched stepwise before measurements and the XPS analysis shows the presence of Zn element uniformly scattered at different depths (Fig. 3c, d), supporting that ZIF-8 nanoparticles were successfully grown in the entire MLDH membrane framework.

The position of ZIF-8 exactly in the pore-like defects of the MLDH framework is crucial to forming the ZIF-8@MLDH membrane for separating Li^+^. The confined ZIF-8 in the framework will impact the transport length only marginally but improve the separation selectivity of the membrane. The site-specific growth of ZIF-8 was promoted by employing ethanol as a solvent for ZIF-8 precursor, and the growth of ZIF-8 nanoparticles was catalyzed by ammonia in ethanol [27, 28]. In order to verify the growth of ZIF-8 nanoparticles at the defects of the MLDH membrane frame rather than between layers, the low-field NMR spectroscopy was applied and detected no peak in the domain of 0.1–10 ms when using ethanol as the probe molecule (Fig. 3b). It implies that ethanol molecule cannot penetrate the interlayer spacing but merely the defects of the MLDH membrane. The reason is that the molecular size of ethanol is larger than the layer spacing of the MLDH membrane and thus excluded from the interlayer. As such, the precursor solution of the ZIF-8 dissolved in and carried by ethanol could only access the defect sites of the framework. During the ZIF-8 formation, the structural stability of the MLDH framework in ethanol and in ethanolic ammonia solution is crucial. In this context, the MLDH framework was first tested by immersion in ethanol and also in an ethanolic ammonia solution for 48 h. The XRD result proved the unchanged interlayer spacing in the MLDH framework after both solution treatments (Figs. S8 and S9). To ensure the in situ growth, the solution of ZIF-8 precursor instead of the dispersion of ZIF-8 seed in ethanol was applied to wet the MLDH framework. The concentration of the precursor solution was regulated to control the growth process. The nucleation kinetics was investigated by monitoring the light transmittance of the ZIF-8 precursor solution at varied concentrations of zinc nitrate (Fig. S10). The molar ratio of zinc nitrate to imidazole was fixed at 3:4 for all solutions. We found that the precursor solution in ethanol remained transparent after 48 h at any concentration of zinc nitrate above 60 g L^−1^, as the solvent ethanol can effectively stabilize such precursors and tune the balance between nucleation and growth of ZIF-8 [28, 29]. Therefore, the concentration of zinc nitrate solution at 60 g L^−1^ as the low boundary was selected to deposit ZIF-8, which turned out to be suitable for growing ZIF-8. Although the surface contact angle was slightly increased, it remains in the hydrophilic range after the growth of hydrophobic ZIF-8 nanoparticles in the hydrophilic MLDH framework (Fig. S11).

### Ion Separation and Stability of the ZIF-8@MLDH Composite Membrane

**Fig. 4:**
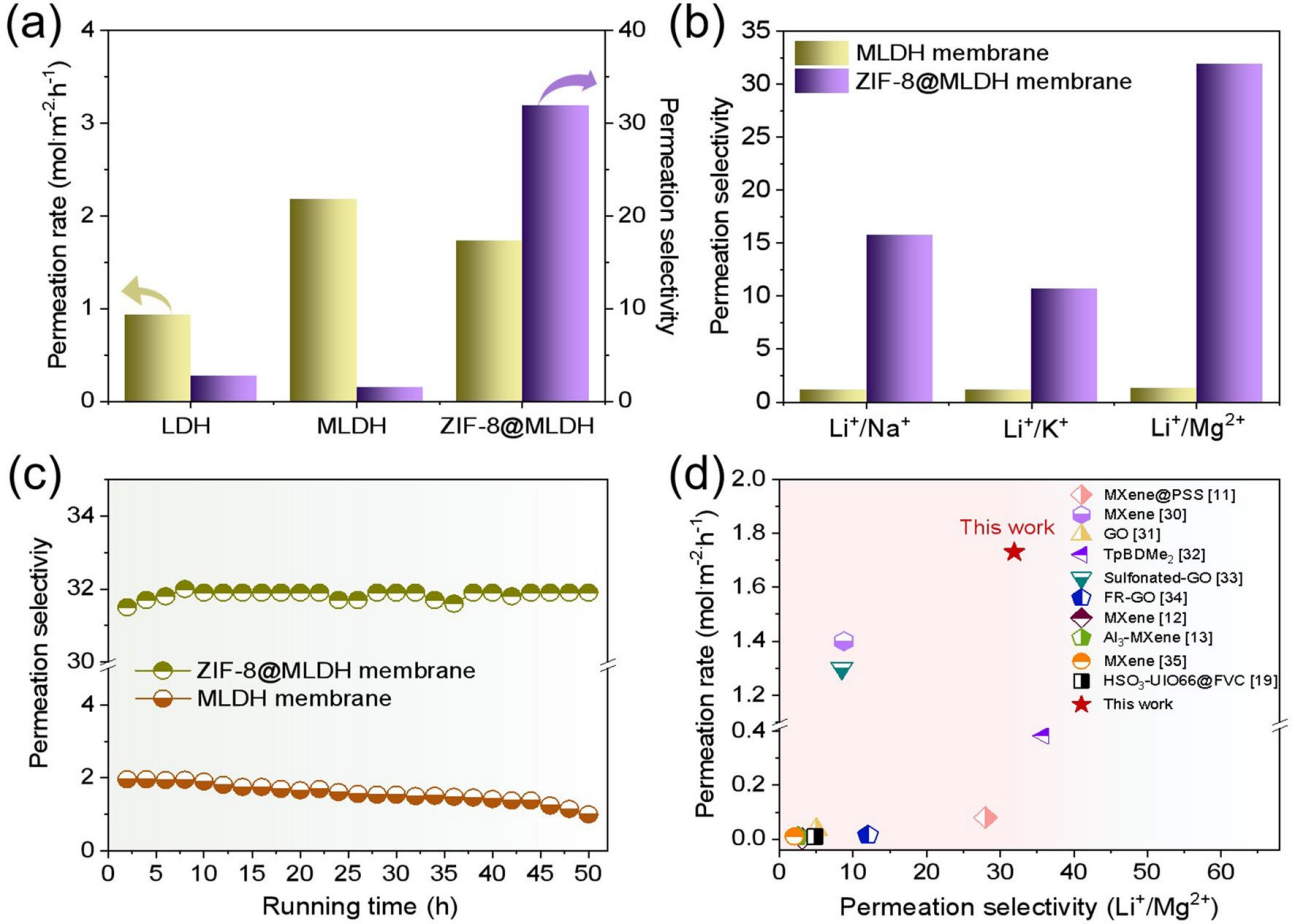
**a** Permeability of different membranes to Li^+^ and the separation performance of Li^+^/Mg^2+^. **b** Comparison of the binary ion selectivity of MLDH and ZIF-8@MLDH composite membranes in 0.2 M LiCl and 0.2 M M^+^Cl_*n*_ (M represents Na, K, and Mg) mixed solutions. **c** Stability tests of the MLDH and ZIF-8@MLDH composite membranes. **d** Permeation rates vs. permeation selectivity of various membranes

The ion permeation behavior was investigated by a homemade U-shaped device (Fig. S12). It is noteworthy that the MLDH membrane had a higher permeation rate for Li^+^ than that of the LDH membrane due to a larger number of vertical transport channels as defects (Fig. 4a). The presence of defect pores in the MLDH lamella increases the number of vertical mass transfer channels and shortens the mass transfer distance that will directly affect the permeability of the membrane (Fig. S13). Since the defects of 16 ± 2 nm in size derived from the etching treatment are dimensionally much larger than ions, the Li^+^/Mg^2+^ selectivity dropped expectedly. Positioning ZIF-8 nanoparticles in the defects naturally narrows the void’s size of frame defects and thus the non-selective nanopores of the MLDH membrane, so that the obtained ZIF-8@MLDH composite membrane carried a much better permeation selectivity of 31.9 for Li^+^/Mg^2+^, and the permeability rate of Li^+^ fell only moderately from 2.18 to 1.73 mol m^−2^ h^−1^ that still defeats the nonetched LDH membrane. This outcome is mainly because ZIF-8 nanoparticles themselves are nanomaterials with a high pore density, so the non-selective pores in the membrane were converted to the needed selective nanochannels.

In addition, in the actual salty lake water system, other cations besides Mg^2+^ will interfere with the extraction of Li^+^. Therefore, we tested the selectivity of Li^+^ to other interfering ions. The results showed that the ZIF-8@MLDH membrane presents a good separation effect on Li^+^/Na^+^ and Li^+^/K^+^, with the selectivity of 15.8 and 10.7, respectively. To note, the separation performance of ZIF-8@MLDH membrane for Li^+^/Mg^2+^ in terms of selectivity is better than that of Na^+^, K^+^, and other interference valence ions, which is in our view related to the strength of ionic charge and the capability of hydration (Fig. 4b). The separation effect of the ZIF-8@MLDH membrane is commonly higher than that of the MLDH membrane, as the ZIF-8@MLDH membrane delivered a specific fast transport capacity for Li^+^. Besides the high permeation rate and selectivity of Li^+^/Mg^2+^, the built-in ZIF-8 in MLDH membranes could advantageously stabilize the framework; at the same time, unlike the pure MOF-based bulk membranes, the in situ growth of ZIF-8 in the confined space within the defects of the MLDH framework is found to be conducive to the stability of ZIF-8 nanoparticles in the aqueous system (Fig. 4c). Such membrane was robust enough under cross-flow conditions and a wide range of pressures (Fig. S14). It supports that the ZIF-8@MLDH membrane can be potentially used in industry-relevant pressure-driven membrane separation processes. Compared with the most advanced 2D materials-based membranes reported recently [30–35], our ZIF-8@MLDH composite membrane presents superior lithium extraction performance both in permeation rate and Li^+^/Mg^2+^ selectivity (Fig. 4d).

It is worth mentioning that the separation performance of the ZIF-8@MLDH membrane could be readily regulated through the thickness of the MLDH membrane (Figs. S15 and S16) controllable between 300 and 1600 nm. The growth of ZIF-8 nanoparticles only expanded the ZIF-8@MLDH membrane thickness marginally, and it is interesting to observe that upon the increase in MLDH membrane thickness, the penetration rate of Li^+^ decreased more slowly than that of Mg^2+^. This intriguing ion-specific drop in the penetration rate of different cations was conducive to improving the selectivity of Li^+^/Mg^2+^. Nevertheless, at a maximum membrane thickness of 1,600 nm, the permeation rate of both Li^+^ and Mg^2+^ declined so significantly to disqualify their practical use. Furthermore, the separation performance is affected to some extent by the concentration of ammonia in the ZIF-8 ethanolic precursor solution during the membrane fabrication. NH_3_ is used to induce the nucleation of the ZIF-8 precursor into nanoparticles, and meanwhile, it also interacts with the MLDH nanosheets. The phase structure of MLDH nanosheets was intrinsically stable in ethanolic ammonia solution, as proven by XRD; their lateral and vertical size within the reaction time changes neither according to the TEM tests (Figs. S17 and S18). Via in-depth analysis, the average pore size of the MLDH nanosheets is found to expand in a slow manner and is positively correlated to the ammonia concentration, indicating an edge-induced weak degradation. This weak-etching effect caused by ammonia is rather beneficial to the composite membrane to adjust its pore size in the nanoscale. At a concentration of 1.33% for the ethanolic ammonia solution, the pores are average 31 nm in size (Fig. S19), which was the maximum nanopore size obtained in this work. The resultant ZIF-8@MLDH membrane accordingly obtained the highest permeation rate and selectivity (Fig. S20). Understandably, larger defects can accommodate larger and more ZIF-8 nanoparticles to regulate the transfer of Li^+^ by their subnanometer channels.

### Theoretical Calculations of Ion Diffusion Mechanism of ZIF-8@MLDH Membranes

**Fig. 5:**
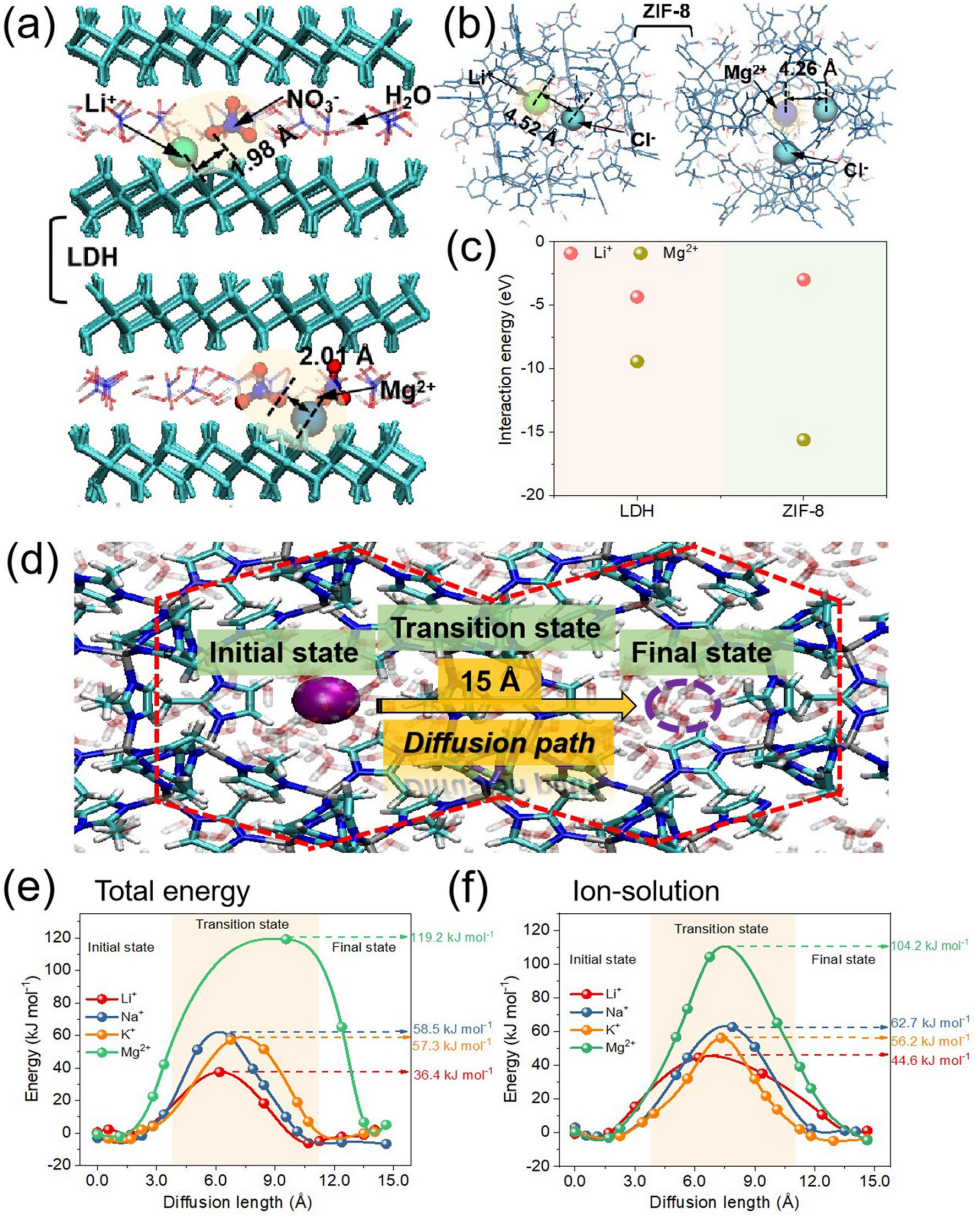
Schematic of selective transport of Li^+^ ion and Mg^2+^ ion in **a** LDH and **b** ZIF-8. **c** Mg^2+^ and Li^+^ ions’ interaction energy of hydrated ionic terminal groups in different channels. **d** Diffusion paths of metal ions in ZIF-8. **e** Total potential energy of metal ions to pass through the ZIF-8 window in the membrane system. **f** Interaction energy between ions and water molecules when metal ions pass through the ZIF-8 window

To better comprehend the diffusion procedure of ions inside the ZIF-8@MLDH membrane, theoretical calculations were carried out. In the MLDH membrane, ions are mainly transported through the interlayer spacing and framework defects of MLDH laminates. After the in situ growth of ZIF-8, the nanoparticles fill in the frame defects in the MLDH membrane; from this moment on, ions are preferentially transported through the pores of ZIF-8. Under molecular dynamics NPT equilibrium, the distribution of Li^+^ and Mg^2+^ in LDH and ZIF-8 is shown in Fig. 5a, b, respectively. Due to the co-existence of anions in the interlayer of LDH, the charge shielding ability of water molecules to ions is limited. To note, along the diffusion process, Li^+^ and Mg^2+^ are combined with anions to maintain the neutrality in charge (the interionic distance is about 2 Å). Therefore, the diffusion of Li^+^ and Mg^2+^ proceeds in the form of salt molecules with both cations and anions. To stress, the anion concentration in ZIF-8 is very low, and thus more difficult for Mg^2+^ and Li^+^ to combine (the interionic distance is greater than 4 Å), so the diffusion is more dominated by the ionic form. The binding energy generated by Mg^2+^ and Li^+^ ions through LDH and ZIF-8 nanochannels was calculated by density functional theory (DFT, Fig. 5c). The binding energy of Li^+^ through the ZIF-8 nanochannel is smaller than that of the LDH nanochannel. Larger binding energy results in retarded ion motion and lowers down ion permeation rate. Therefore, the transmission rate of Li^+^ within ZIF-8 is greater than that between LDH layers. In comparison, the calculation shows that the binding energy of Mg^2+^ in the ZIF-8 channel is stronger than that between LDH layers, indicating the more unfavored transport of Mg^2+^ in the ZIF-8 channel than between LDH layers. These comparisons show that the Li^+^ penetration rate and selectivity of Li^+^/Mg^2+^ in the ZIF-8@MLDH membrane are logically higher than those in the LDH membrane, which is consistent with the experimental results (Fig. 4a).

Metal ions of Li^+^, Na^+^, K^+^, and Mg^2+^ have different diffusion rates when transporting through ZIF-8. We simulated their diffusion process from one ZIF-8 cavity to an adjacent cavity through a ZIF-8 window by molecular dynamics (MD) simulations. The set diffusion path is shown in Fig. 5d, and the diffusion distance was set as 15 Å. Figure 5e depicts the change of the total potential energy of the system when four ions pass through the window. The height of the barrier represents the energy that ion diffusion needs to overcome; obviously the higher the barrier is, the more difficulty the ions diffuse. The potential barriers of Li^+^, Na^+^, K^+^, and Mg^2+^ passing through the window region are 36.4, 58.5, 57.3, and 119.2 kJ mol^−1^, respectively. It is consistent with the experimental results that the selectivity drops in the sequence of Li^+^/Mg^2+^  > Li^+^/Na^+^  > Li^+^/K^+^ (Fig. 4b). It should be noted that the strength of the total barrier is jointly affected by the interaction between ions and water molecules, the deformation energy of ZIF-8 and the interaction between ions and ZIF-8. Here, the interaction energy between ions and water molecules is more dominant (Fig. 5f).

To further explore the difference in dehydration energy, the dehydration process of the four ions was discussed in detail here. As shown in Fig. 5f, the width of the dehydration energy barrier can reflect the radius of hydrated ions laterally, because hydrated ions with a large radius need to undergo a larger range of dehydration when passing through the ZIF-8 window, and the ion dehydration will lead to changes in energy. The radius of hydrated ions can be approximated by the radial distribution function *g*(r) of ions and the adjacent water molecules, as shown in Fig. S21a. As seen, the second peak of Mg^2+^ is sufficiently strong, indicating that Mg^2+^ forms a stable ion structure of double-layer hydration. As such, the Mg-H_2_O distance reaches 4.2 Å, which is large enough to seriously hinder the permeability of hydrated Mg^2+^. To note, the stable double-layer of hydration is associated with the small radius, large charge, and strong Coulombic interaction of Mg^2+^ with adjacent water molecules. While Li^+^, Na^+^, and K^+^ are monocharged, they carry flatter second peaks than Mg^2+^, resulting in weak binding to the second layer of water. Therefore, it is the first layer of water molecules (Li^+^-H_2_O, Na^+^-H_2_O, and K^+^-H_2_O at distances of 2.0, 2.4, and 2.8 Å, respectively) that play a major obstructive role in the permeability of these monocharged ions. Obviously, the binding radius of Li^+^, Na^+^, and K^+^ with one water molecule layer is smaller, and it is easier to pass through the window than Mg^2+^. In addition, according to the simulation results (Fig. S21b), it was found that when ions moved from the ZIF-8 cavity to the window, Li^+^ maintained a four-coordination structure with water molecules and could pass through the window without dehydration. Na^+^ and K^+^ have a six-coordination structure with water molecules in the ZIF-8 cavity and become a four-coordination structure when passing through the ZIF-8 window, meaning that a part of the water molecules needs to be removed to pass through the window. Therefore, Na^+^ and K^+^ require higher energy than Li^+^ when passing through the ZIF-8 window, so the penetration rate of Li^+^ is greater than that of Na^+^ and K^+^. It is worth mentioning that the larger ionic radius of K^+^ than Na^+^ weakens the interaction between K^+^ and adjacent water molecules and reduces dehydration energy thus K^+^ is slightly easier to diffuse than Na^+^. In short, the permeability rate of ions follows the series of Li^+^  > K^+^  > Na^+^  > Mg^2+^, which is consistent with the experimental results.

## Conclusion

In summary, the internal defects of the MLDH framework were constructed by alkaline etching treatment of LDH nanosheets and engineered through in situ growth of ZIF-8 nanoparticles. The as-prepared ZIF-8@MLDH membranes make good use of the confined ZIF-8 nanoparticles in the composite membrane to boost the penetration rate of Li^+^. The in situ growth of ZIF-8 nanoparticles was found to reduce the defect aperture in the framework and improve the ion selectivity as well as the structural stability of the entire membranes. The Li^+^/Mg^2+^ selectivity of these membranes reaches as high as 31.9 with a large Li^+^ permeation rate of 1.73 mol m^−2^ h^−1^. They also present enhanced separation performance for Li^+^/Na^+^ and Li^+^/K^+^. Through simulation and chemical environment analysis, the confined channel has been demonstrated as a synergistic mechanism of ion infiltration. The synthetic strategy of the ZIF-8@MLDH membrane adopted in this study provides new insights into the preparation of 2D lamellar membranes with high selectivity, permeability, and stability. The research results will also assist the design of efficient ion separation membranes, especially lithium extraction membranes.

### Supplementary Information

Below is the link to the electronic supplementary material.

Supplementary file1 (PDF 1410 KB)
