# Supplementary material for: Engineer Nanoscale Defects into Selective Channels: MOF-Enhanced Li+ Separation by Porous Layered Double Hydroxide Membrane
Source: Nanomicro Lett. 2023 Jun 7;15:147. doi: 10.1007/s40820-023-01101-w (PMC10247908; doi:10.1007/s40820-023-01101-w)
Supplement: Supplementary file 1 — Supplementary file1 (PDF 1410 KB) [file 40820_2023_1101_MOESM1_ESM.pdf]

Supporting Information for

# Engineer Nanoscale Defects into Selective Channels: MOF-Enhanced $Li^+$ Separation by Porous Layered Double Hydroxide Membrane

Yahua Lu[1, 3], Rongkun Zhou[2], Naixin Wang[1,] *, Yuye Yang[1], Zilong Zheng[2], Miao Zhang[3], Quan-Fu An[1,] *, Jiayin Yuan [3,] *

[1]Beijing Key Laboratory for Green Catalysis and Separation, College of Environmental and Energy Engineering, Beijing University of Technology, Beijing 100124, P. R. China

[2]Faculty of Materials and Manufacturing, Beijing University of Technology, Beijing 100124, P.R. China

[3]Department of Materials and Environmental Chemistry, Stockholm University, Stockholm 10691, Sweden

*Corresponding authors. E-mail: wangnx@bjut.edu.cn (Naixin Wang), anqf@bjut.edu.cn (Quan-Fu An), jiayin.yuan@mmk.su.se (Jiayin Yuan)

## S1 Supplementary Experimental Procedures

### S1.1 Ion Permeation Tests

First, the membrane sealed by the rubber gasket was placed in the middle of the homemade U-shaped device. Then, 0.2 M ion-containing aqueous solution was placed on one side of the U-shaped device (the side directly in contact with the membrane). The other side was filled with an equal volume of DI water (resistance: 18.2 MΩ) and stirred to prevent concentration polarization. The results were collected every 5 mins. The calculation method of the ion concentration from ion conductivity was based on the previous work [S1-S3]. The calculation formula for theion permeation rate is as follows:

$$P_{\mathrm{i}} = \frac{(C_1 - C_0) * V}{(A * t)} \tag{S1}$$

$C_1$ represents the current ion concentration ($mol\ L^{-1}$), $C_0$ represents the previous ion concentration ($mol\ L^{-1}$), $V$ represents the volume of the aqueous solution ($mL$), $A$ represents effective membrane area ($m^2$), $t$ represents an interval of test time ($h$).

In the ion mixture, the ion permeation rate was obtained by the ICP-MS technique. The ion permeation rate calculation formula is as follows:

$$P_{M_{\mathrm{i}}} = \frac{(\rho_1 - \rho_0) * V}{(A * t)} \tag{S2}$$

$$P_i = \frac{P_{M_i}}{M_i} \tag{S3}$$

Here, $P_{Mi}$ represents the mass permeation rate ($g\ m^{-2}\ h^{-1}$). $P_i$ represents the mol permeation rate ($mol\ m^{-2}\ h^{-1}$). $\rho_1$ represents current ion mass concentration ($ppm$), $\rho_0$ represents previous ion mass concentration ($ppm$), $V$ represents the volume of the aqueous solution ($mL$), $M_i$ represents atomic molar mass ($mg\ mol^{-1}$), $A$ represents effective membrane area ($m^2$), $t$ represents the interval of test time ($h$).

The formula for real ion permeation selectivity ($Li^+/Na^+$, $Li^+/K^+$, and $Li^+/Mg^{2+}$) is shown below:

$$SF_{mass} = \frac{P_{M1}}{P_{M2}} \tag{S4}$$

$$SF_{mol} = \frac{P_{M1} * M_2}{P_{M2} * M_1} = SFmass * \frac{M_2}{M_1} \tag{S5}$$

## S1.2 Evaluation of Water Permeability

Water was used as the feed, which was pressurized with a plunger pump. The water was recycled back to the feed tank while permeate water was collected. The pure water permeance was calculated from real-time measurements of water flux ($J_w$), corresponding to the volume of collected water. Filtration characteristics, including water permeability, were determined under steady-state conditions. They were calculated according to the following equations:

$$J_w = \frac{V}{A * t * \Delta P} \tag{S6}$$

Where $V$ is the volume of permeate ($L$), $A$ is the effective membrane area ($m^2$), $t$ is the permeation time ($h$), and $\Delta P$ is the transmembrane pressure ($bar$).

## S1.3 Relationship between Permeability and Mass Transfer Path

We compared the water permeability of the LDH membrane and MLDH membrane, that is, the relationship between permeability and mass transfer path. It was found that the water permeability of the MLDH membrane was three times higher than that of the LDH membrane because the etched defects in the MLDH membrane shortened the mass transfer path of the MLDH membrane. The water permeability calculation formula was as follows:

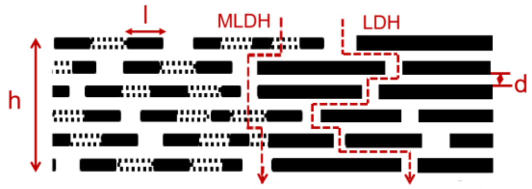


**Scheme S1** Mass transfer path of MLDH and LDH membrane

$$A = l * \frac{h}{d} \quad \text{(S7)}$$

*A* represents the mass transfer path length (*nm*), *h* represents the membrane thickness (*nm*), *l* represents the diameter of lamella (*nm*), *d* is the interlayer spacing (*nm*) [S4].

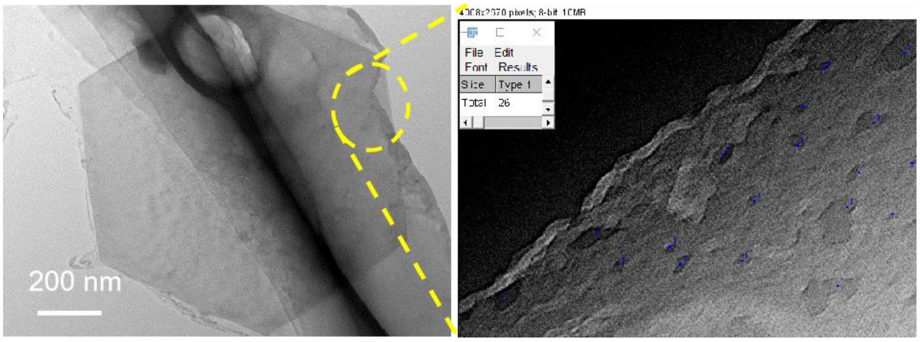


**Scheme S2** The number of holes in MLDH lamellae calculated by Image J

For the etched MLDH nanosheet, Image J software is used to calculate the number of holes in the lamella. As shown in the figures above, the number of holes in the calculated area is 26, and the area size accounts for 1/32 of the entire lamella. As mentioned above, the etched aperture size is about 16 nm. Therefore, in the whole lamella, the diameter of the etched MLDH plate is about 340 nm.

The mass transfer path length of the LDH membrane and MLDH membrane are calculated as follows:

$$A_{LDH} = 1000 * \frac{1200}{0.34} = 3529\mu m$$

$$A_{MLDH} = 340 * \frac{1220}{0.34} = 1220\mu m$$

As shown in the calculated results, the mass transfer path length of the LDH membrane is three times longer than that of the MLDH membrane, which is consistent with the results of water permeability.

## S1.4 Characterization

Microstructures and elemental distribution analysis of LDH membrane, MLDH membrane, and nanosheets were achieved through Field Emission Scanning Electron Microscopy (FESEM, Hitachi SU8100) and Energy-Dispersive X-ray spectroscopy (EDX, Oxford EDS, with INCA software), respectively. The further characterization of the LDH and MLDH nanosheets by electron microscopy Transmission Electron Microscope (Hitachi JEM-2100F). The lamellar LDH and MLDH nanosheet surface images were captured via the Atomic Force Microscope (AFM, Bruker Multimode 8) under tapping mode at ambient conditions. Samples were treated on the monolayer mica and dried under vacuum conditions for further characterization. The lateral size

distribution of lamellar LDH and MLDH nanosheets was achieved by counting AFM images of different regions. The structure of LDH and MLDH membrane was obtained through X-ray Diffraction (XRD, Rigaku Smart Lab X-Ray Diffractometer) using Cu Kα radiation from $2^o$ to $40^o$ with a scanning rate of $10^o$ $min^{-1}$. The zeta potential of the MLDH nanosheet solution was achieved from the dynamic laser scattering (DLS, Malvern Nano ZS) in a polystyrene cuvette. The inner chemistry of the ZIF-8@MLDH membrane was characterized through X-ray Photoelectron Spectroscopy (XPS) ESCALAB 250xi spectrometer (Thermo Fisher Scientific) with monochromatic Al-Kα radiation (1486.6 eV) under a pressure of $2\times10^{-9}$ Torr. LF-NMR measurements were performed on LDH membrane and MLDH membrane and ZIF-8@MLDH membrane on a 22.4 MHz NMR analyzer (A NMI20-Analyst NMR analyzer, Suzhou Niumag Analytical Instrument Corporation, China). Ion concentration was achieved through Inductively Coupled Plasma-Mass Spectrometry (ICP-MS, Thermal Fisher Scientific, iCAP RQ) with Argon plasma as the excitation source, and scandium is used as the standard internal element.

### S1.5 MD Simulations

The structures of different cations combined with LDH and ZIF-8 materials in an aqueous solution were obtained by MD simulations. The simulation boxes for cations in LDH consist of a 12×8×2 periodic supercell of LDH, containing two metal-hydroxide layers, anions, cations, and water molecules. The number of water molecules per interlayer was set to 30 to adjust the interlayer distance to 8.5 Å which is the same as the experimental value. The quantity ratio of $Mg^{2+}$ ion to $Al^{3+}$ ion is 3:1. And the number of nitrate ions ($NO_3^-$) was set to 12 per interlayer to neutralize the negative charge of LDH. The interatomic potentials of LDH are described based on the CLAYFF force field [S5]. The simulation boxes for cations in ZIF-8 were performed on a 1×1×1 unit cell of ZIF-8, containing 70 water molecules. AMBER-99 force field was employed to describe the interatomic potentials of ZIF-8 [S6]. The number of $Cl^-$ is 1 or 2, which depends on whether the cation is $Li^+$, $Na^+$, $K^+$, or $Mg^{2+}$. For the $H_2O$ behavior, the SPC/E water model is used. To identify the variation of total energy and interaction potential between cation and aqueous solution, we set the transport rates of cations from point 1 to point 2 shown in Fig. 5c to 7.5 Å/ns.

All mixture systems were equilibrated by a canonical ensemble (NVT) MD simulation for 0.5 ns at 300 K and followed by constant-pressure-constant-temperature (NPT) MD simulations for 8 ns at 1 atm. All MD simulations were performed using the GROMACS (version 2018.4) [S7, S8] software package. The temperature was controlled by a Nose-Hoover Langevin (NHL) thermostat and the pressure was controlled by a Berendsen thermostat [S9, S10]. Periodic boundary conditions have been used. Particle-Mesh Ewald (PME) electrostatics were applied to calculate the long-range electrostatic interactions. The short-range electrostatic and van der Waals (vdW) cutoff distances were both set to 0.8 nm, respectively. The time step for all MD simulations was taken as 2 fs. To calculate the distances between different cations and the surrounded water molecules,

the structures in the last 4 ns were used for radial distribution function (RDF) analysis.

### S1.6 DFT Calculations

The binding energies (Eb) between cations ($Li^+$, $Mg^{2+}$) and separator materials (LDH, ZIF-8) were calculated by Vienna Ab-initio Simulation Package (VASP) [S11] code with Perdew, Burke, and Ernzerhof (PBE) generalized gradient approximation (GGA) [S12] for exchange-correlation. The Brillouin zone was sampled by a set of 1×1×1 K-mesh. The cut-off energy for basic functions was 550 eV. The tolerance of total energy convergence is less than $10^{-5}$ eV. Owing to the high concentration of $NO_3^-$ in LDH, $Li^+$, and $Mg^{2+}$ ions combine with one and two $NO_3^-$ ions respectively in the diffusion process. Thus, the interaction of cations with LDH can be affected by $NO_3^-$.

The $E_b$ between $LiNO_3$ and LDH is as follows:

$$E_b = E[LiNO_3(LDH)] - E(LDH) - E(LiNO_3) \quad \text{(S8)}$$

The $E_b$ between $Mg(NO_3)_2$ and LDH is as follows:

$$E_b = E[\mathrm{Mg(NO_3)_2(LDH)}] - \mathrm{E(LDH)} - \mathrm{E(Mg(NO_3)_2)} \quad \text{(S9)}$$

Where *E[LiNO₃ (LDH)]* and *E[Mg (NO₃)₂ (LDH)]* are the total energy of $LiNO_3$ and Mg $(NO_3)_2$ combined with LDH respectively. $E(LiNO_3)$, $E(Mg(NO_3)_2)$ and *E(LDH)* represent the energies of $LiNO_3$, Mg $(NO_3)_2$ and LDH.

Due to the low concentration of $Cl^-$ ion, thus, the charge shielding effect of cations by anions during the diffusion process is relatively weak.

The $E_b$ between cation and ZIF-8 is as follows:

$$E_b = \mathrm{E[cation\ (ZIF\text{-}8)]} - E(ZIF\text{-}8) - E(\mathrm{cation}) \quad \text{(S10)}$$

Where *E[cation(ZIF-8)]* is the total energy of $Li^+$ or $Mg^{2+}$ combined with ZIF-8. *E(ZIF-8)* is the energy of ZIF-8. *E(cation)* is the energy of cation ($Li^+$ or $Mg^{2+}$). The binding energies between a cation and a water molecule in the first and second solvation shell were performed at B3LYP/6-31G(d) level using Gaussian 09 program package [S13], and are calculated by randomly selecting five segments from the trajectory obtained by MD simulation.

The $E_b$ between cation and $H_2O$ is as follows:

$$E_b = E[cation(H_2O)] - E(H_2O) - E(cation) \quad \text{(S11)}$$

Where $E(H_2O)$ is the energy of one $H_2O$.

## S2 Supplementary Figures

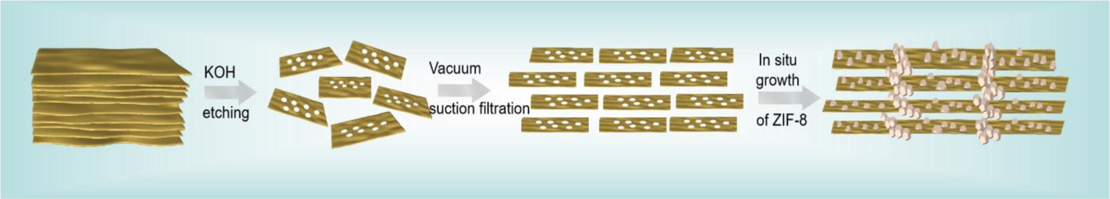


**Fig. S1** Schematic of the preparation process of the ZIF-8@MLDH membrane

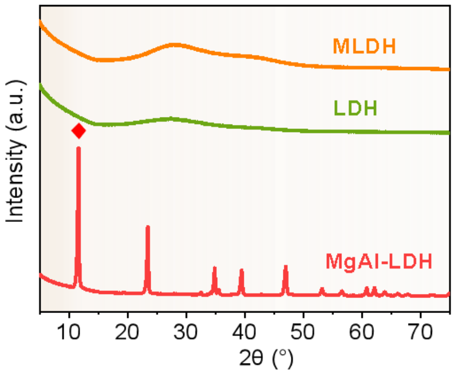


**Fig. S2** XRD diagrams of LDH nanosheets before (green line) and after etching (orange line) and MgAl-LDH nanoparticles (red line)

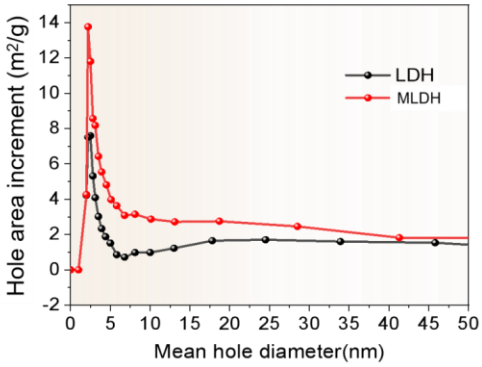


**Fig. S3** Pore size distribution plots of LDH nanosheets before and after etching

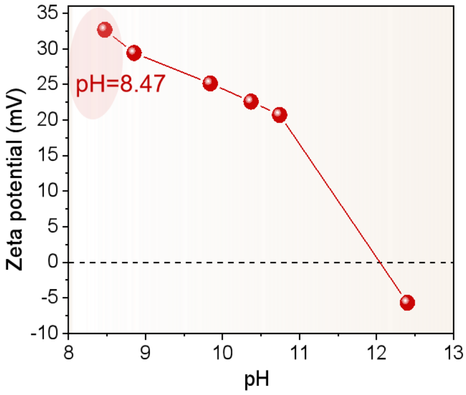


**Fig. S4** Zeta potential of MLDH dispersion at various pH values

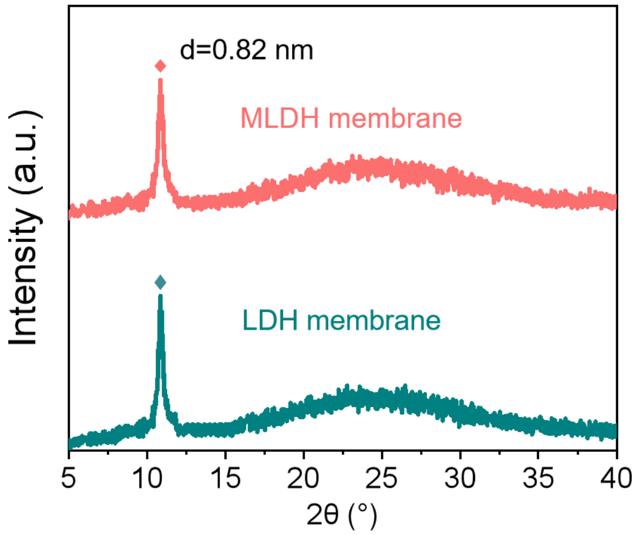


**Fig. S5** XRD diagrams of LDH membrane and MLDH membrane

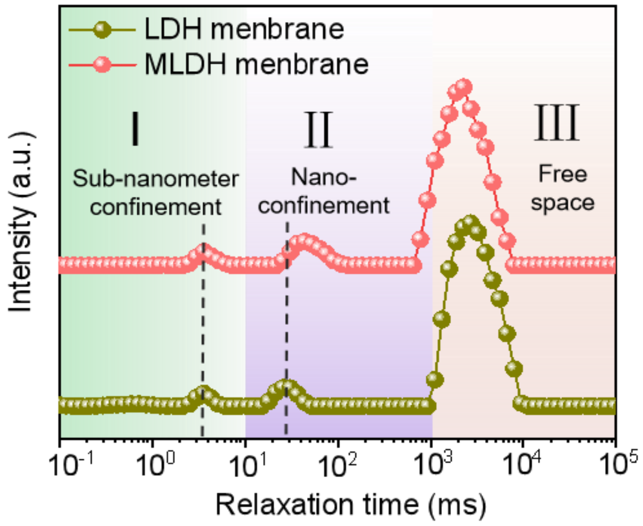


**Fig. S6** $^{1}H$ time-domain nuclear magnetic resonance spectra of LDH and MLDH membrane with water as the probe molecule

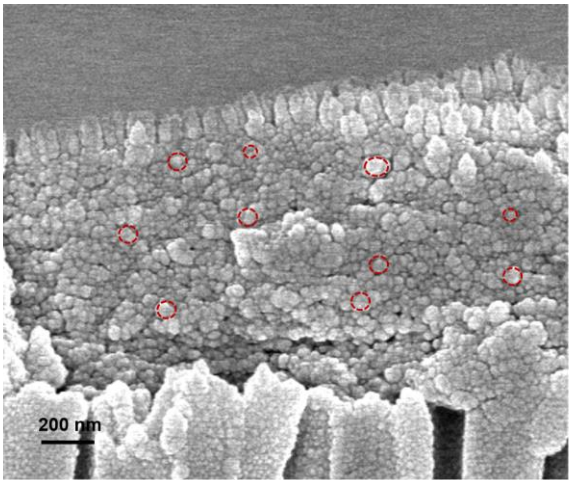


**Fig. S7** High-magnification SEM cross-section of the ZIF-8@MLDH membrane

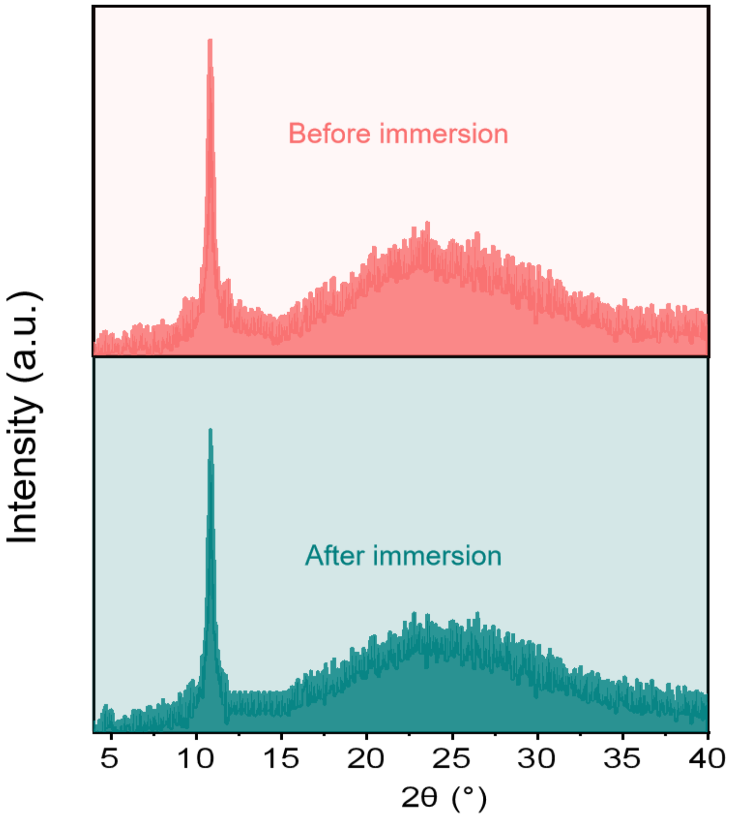


**Fig. S8** XRD diagrams of MLDH membrane before and after soaking in ethanol for 48 h

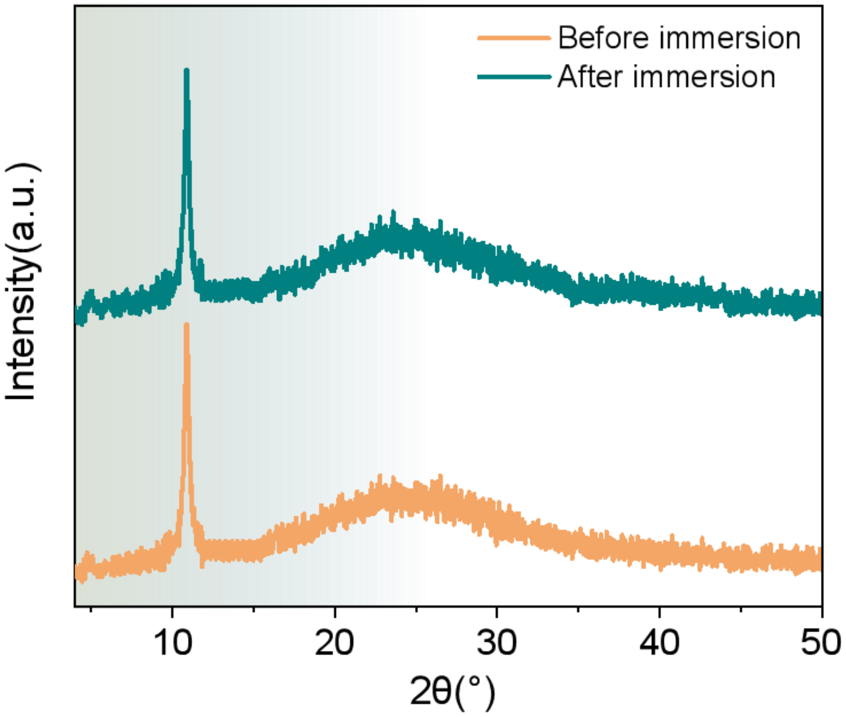


**Fig. S9** XRD diagrams of MLDH membrane before and after soaked in 75:1 proportion of ethanol and ammonia solution for 48 h

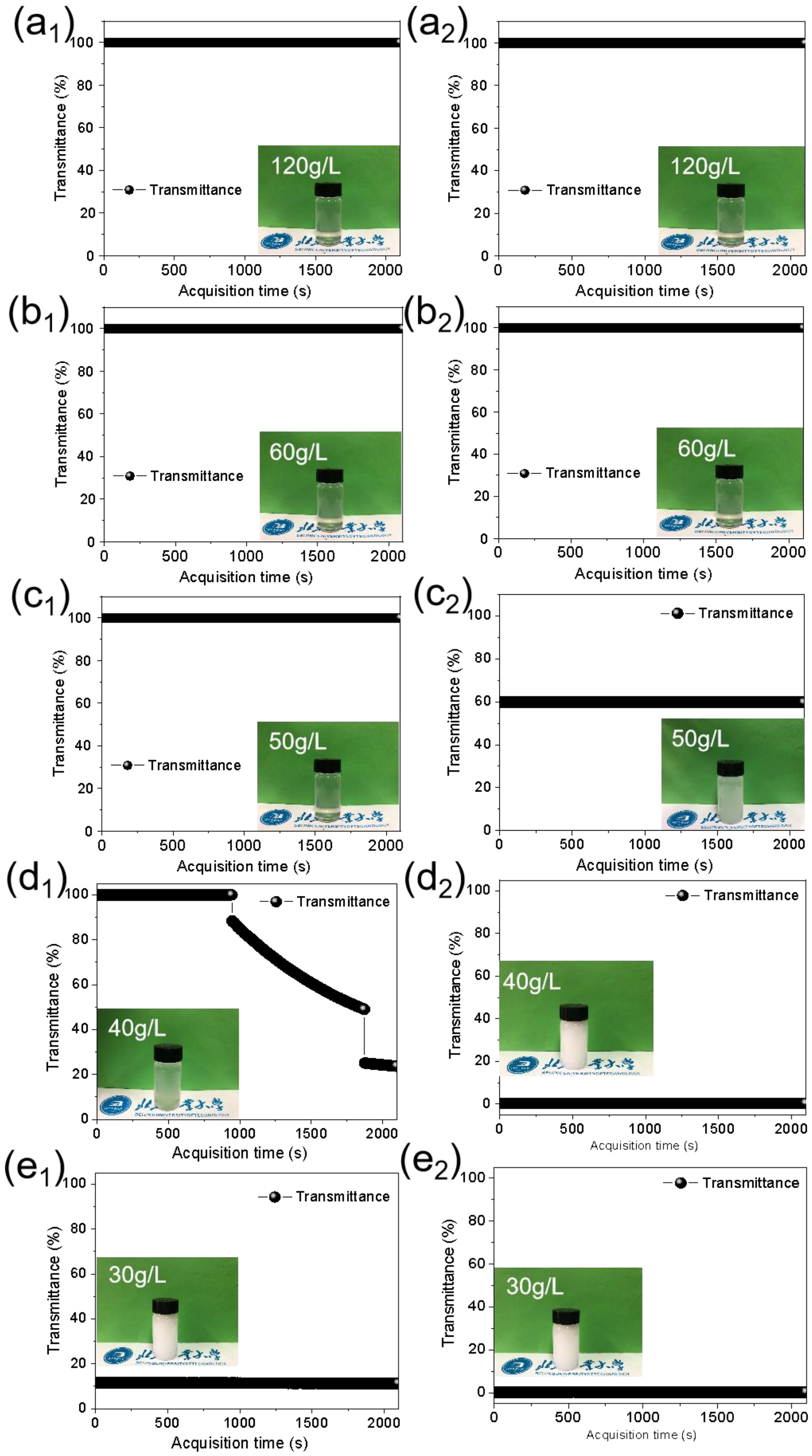


**Fig. S10** Light transmittance of ZIF-8 precursor solutions at different concentrations of zinc nitrate at initial time $\mathbf{a_1}$ - $\mathbf{e_1}$ and standing after 48 h $\mathbf{a_2}$ - $\mathbf{e_2}$. The concentration ratio of zinc nitrate and dimethyl imidazole was 3:4

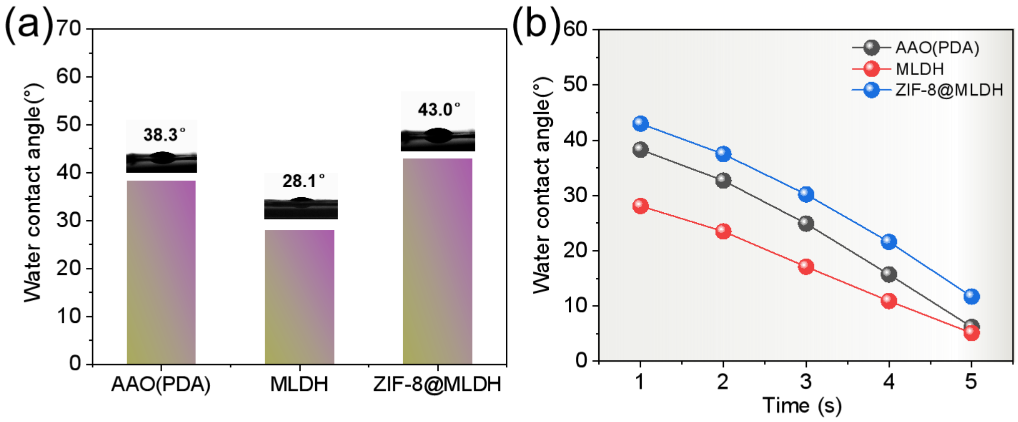


**Fig. S11 a** Static water contact angle and **b** Dynamic water contact angle for different membranes

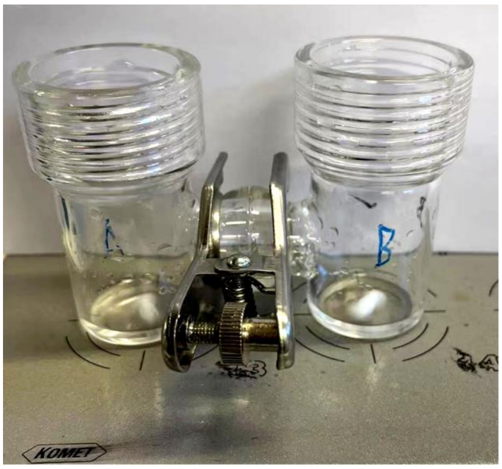

**Fig. S12** A self-made U-shaped device to investigate the ion permeation behavior

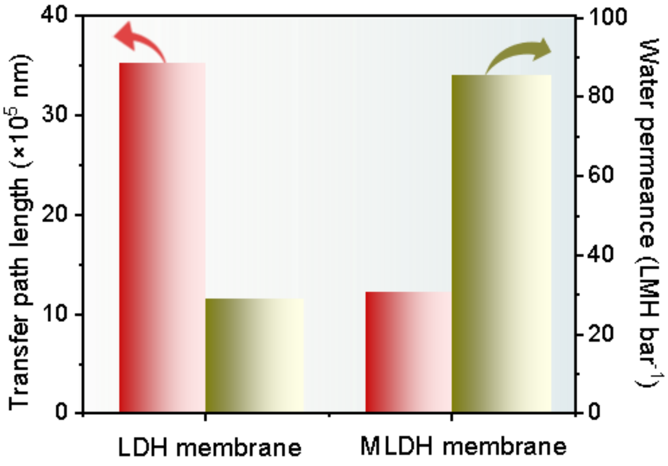


**Fig. S13** The relationship between transfer path length and water permeability for LDH and MLDH membrane (The experimental results are in agreement with the theoretical results)

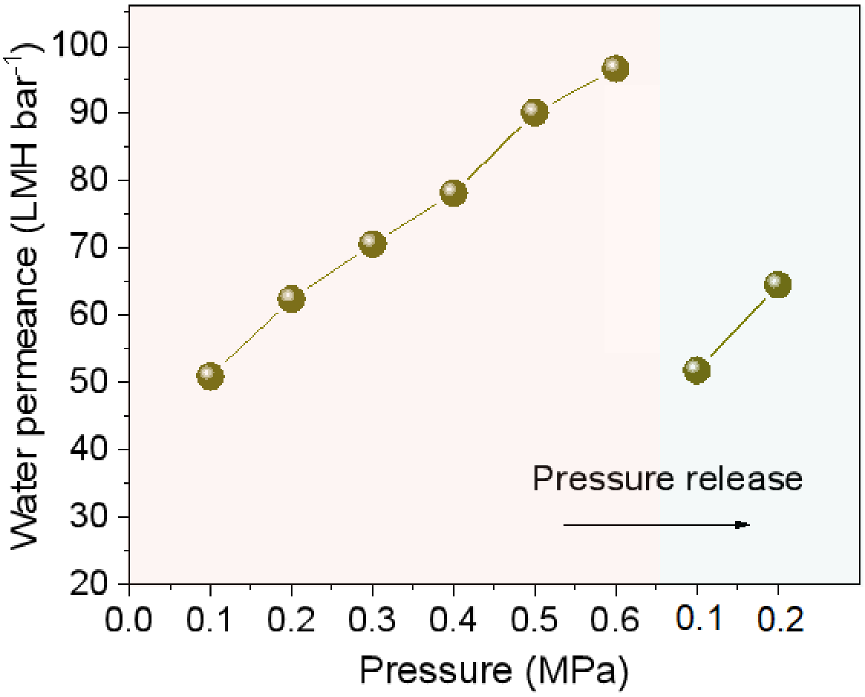


**Fig. S14** Effect of operating pressure on the stability of ZIF-8@MLDH composite membrane in a cross-flow filtration device

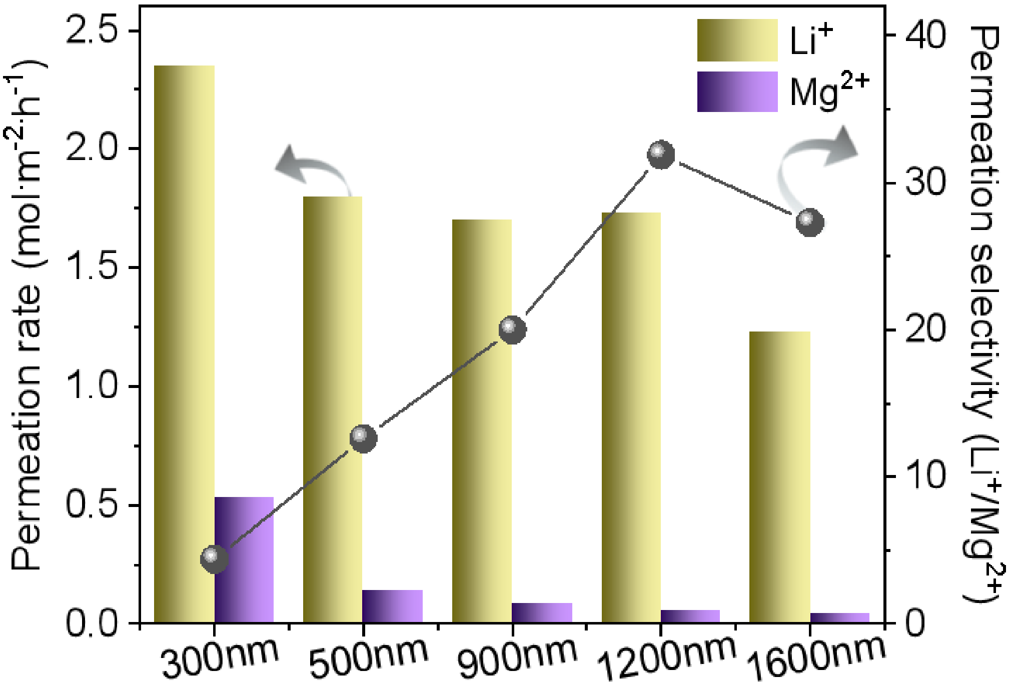


**Fig. S15** Separation performance of ZIF-8@MLDH membranes with different thickness

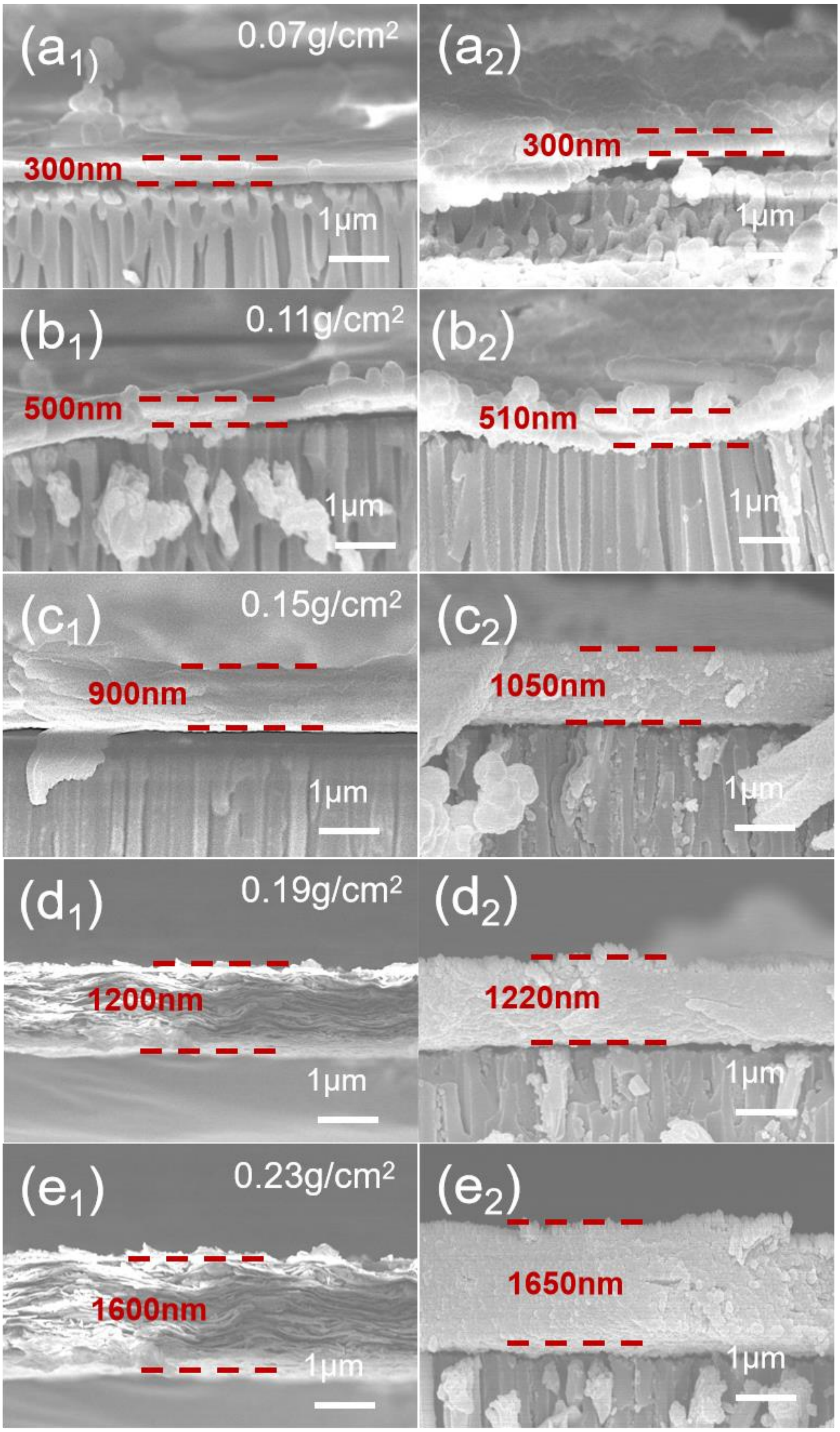


**Fig. S16** Membrane thickness with different MLDH loading **$a_1$ - $e_1$** Before and **$a_2$ - $e_2$** After growth of ZIF-8

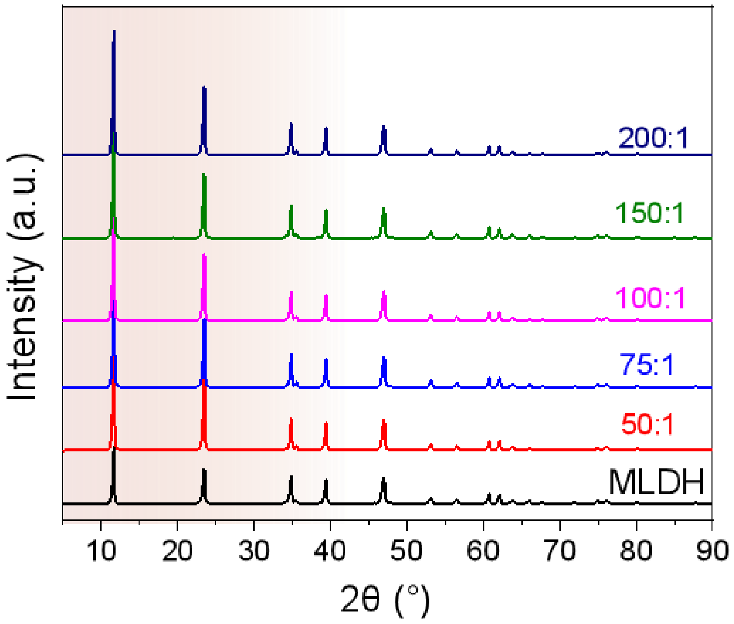


**Fig. S17** XRD diagrams of MLDH nanosheets soaked in different proportions of ethanol and ammonia solution for 12 h

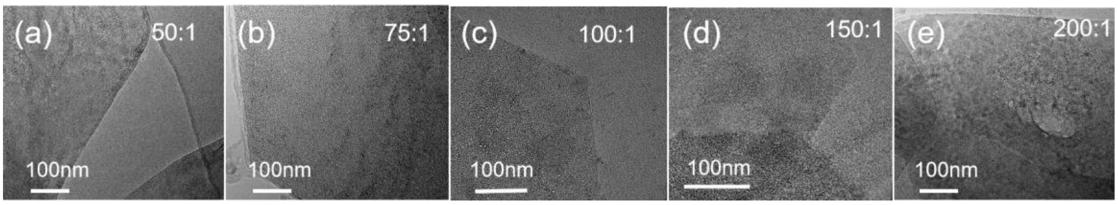


**Fig. S18** TEM imagines of dried MLDH nanosheets soaked in different proportions of ethanol and ammonia solution for 12 h

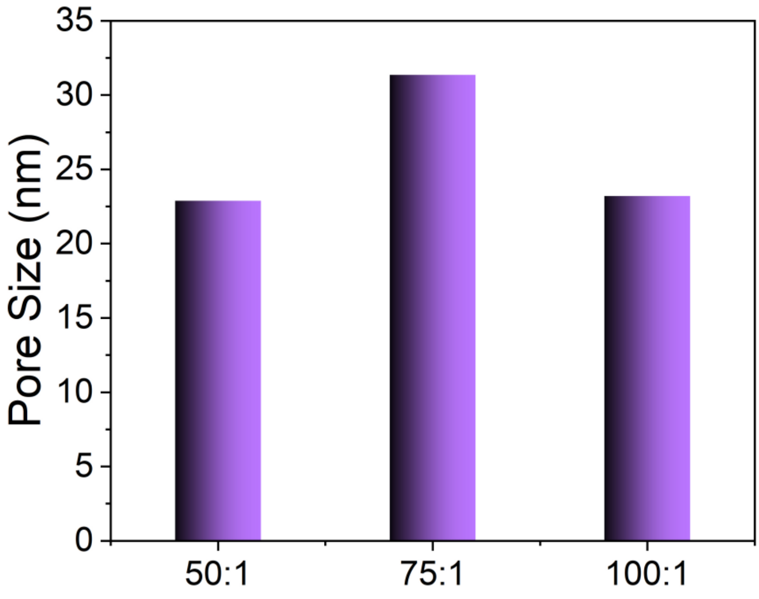


**Fig. S19** Pore size of MLDH nanosheets soaked in different proportions of ethanol and ammonia solution for 12 h

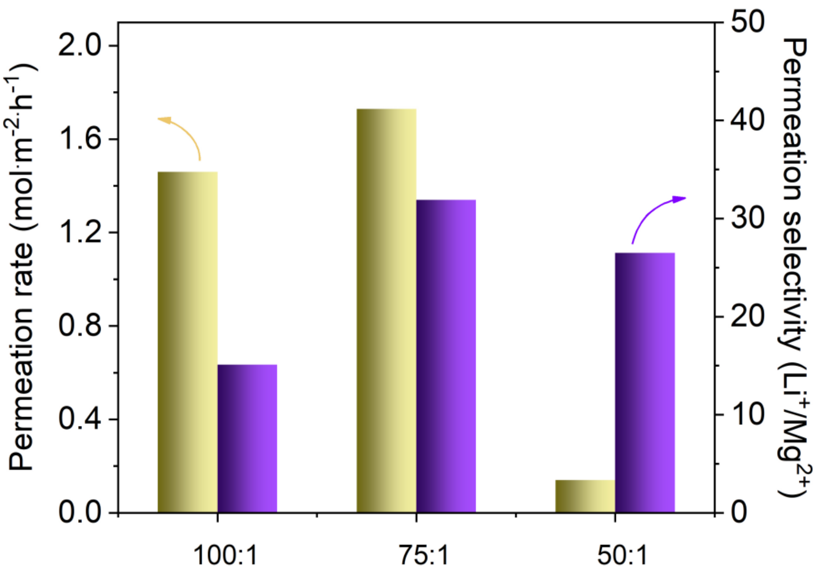


**Fig. S20** Separation performance of ZIF-8@MLDH membrane with different ethanol -ammonia ratio in the precursor solution

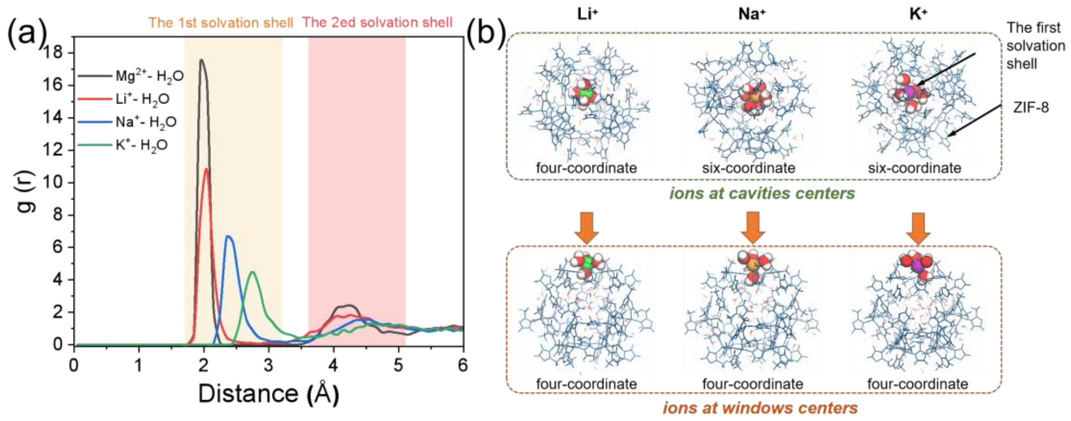


**Fig. S21 a** Radial distribution diagram of metal ions with adjacent water molecules. The g(r) represents the probability to find an oxygen atom of the water molecule in a shell with a given thickness at the distance r of the central ion chosen as a reference point. The higher peak of g(r) represents the larger density of oxygen atoms at the distance r. **b** Coordination number changes of ions with water molecules in the ZIF-8 cavity and at the ZIF-8 window

## Supplementary References

[S1] Z. Lu, Y.Y. Wei, J.J. Deng, L. Ding, Z.K. Lin et al., Self-crosslinked MXene ($Ti_3C_2Tx$) membranes with good antiswelling property for monovalent metal ion exclusion. ACS Nano **13**, 10535 (2019). https://doi.org/10.1021/acsnano.9b04612

[S2] L. Ding, L.B. Li, Y.C. Liu, Y. Wu, Z. Lu et al., Effective ion sieving with $Ti_3C_2T_x$ MXene membranes for production of drinking water from seawater. Nat. Sustain. **3**, 296 (2020). https://doi.org/10.1038/s41893-020-0474-0

[S3] Z. Lu, Y. Wu, L. Ding, Y.Y. Wei, H.H. Wang, A lamellar MXene ($Ti_3C_2T_x$)/PSS composite membrane for fast and selective lithium-ion separation. Angew. Chem. Int. Ed. **133**, 22439 (2021). https://doi.org/10.1002/anie.202108801

[S4] G.Y. Dong, J. Hou, J. Wang, Y. Zhang, V. Chen et al., Enhanced $CO_2/N_2$ separation by porous reduced graphene oxide/Pebax mixed matrix membranes. J. Membr. Sci. **520**, 860 (2016). https://doi.org/10.1016/j.memsci.2016.08.059

[S5] R.T. Cygan, J. Liang, A.G. Kalinichev, Molecular models of hydroxide, oxyhydroxide, and clay phases and the development of a general force field. J. Phys. Chem. B **108**, 1255 (2004). https://doi.org/10.1021/jp0363287

[S6] E.J. Sorin, V.S. Pande, Exploring the helix-coil transition *via* all-atom equilibrium ensemble simulations. Biopsy. J. **88**, 2472 (2005). https://doi.org/10.1529/biophysj.104.051938

[S7] B. Hess, C. Kutzner, D.v.d. Spoel, E. Lindahl, GROMACS 4: Algorithms for highly efficient, load-balanced, and scalable molecular simulation. J. Chem. Theory. Comput. **4**, 435 (2008). https://doi.org/10.1021/ct700301q

[S8] S. Pronk, S. Páll, R. Schulz, P. Larsson, P. Bjelkmar et al., GROMACS 4.5: a high-throughput and highly parallel open source molecular simulation toolkit. Bioinformatics **29**, 845 (2013). https://doi.org/10.1093/bioinformatics/btt055

[S9] H.J.C. Berendsen, J.P.M. Postma, W.F. van Gunsteren, A. DiNola, J.R. Haak, Molecular dynamics with coupling to an external bath. J. Chem. Phys. **81**, 3684 (1984). https://doi.org/10.1063/1.448118

[S10] A.A. Samoletov, C.P. Dettmann, M.A.J. Chaplain, Thermostats for “slow” configurational modes. J. Stat. Phys. **128**, 1321 (2007). https://doi.org/10.1007/s10955-007-9365-2

[S11] G. Kresse, J. Furthmüller, Efficient iterative schemes for ab initio total-energy calculations using a plane-wave basis set. Phy. Rev. B **54**, 11169 (1996). https://doi.org/10.1103/physrevb.54.11169

[S12] J.P. Perdew, K. Burke, M. Ernzerhof, Generalized gradient approximation made simple. Phys. Rev. Lett. **77**, 3865 (1996). https://doi.org/10.1103/physrevlett.77.3865

**[S13]** J. Hiscocks, M.J. Frisch, Gaussian 09, IOps Reference. Gaussian, 2009.
